# Effect of Photocatalyst Aggregation on Photocatalytic Reaction Rate Concentration Dependence

**DOI:** 10.3390/ma19122504

**Published:** 2026-06-10

**Authors:** Vanya Lilova, Emil Lilov, Svetlozar Nedev, Stephan Kozhukharov, Christian Girginov, Veronica Nemska

**Affiliations:** 1 Department of Physics, University of Chemical Technology and Metallurgy, 8 Kliment Ohridski Blvd., 1797 Sofia, Bulgaria; emo.lilov@uctm.edu (E.L.); nedev@uctm.edu (S.N.); s.kozhukharov@uctm.edu (S.K.); 2 Department of Physical Chemistry, University of Chemical Technology and Metallurgy, 8 Kliment Ohridski Blvd., 1756 Sofia, Bulgaria; girginov@uctm.edu; 3 Department of Biotechnology, University of Chemical Technology and Metallurgy, 8 Kliment Ohridski Blvd., 1756 Sofia, Bulgaria; vnemska@uctm.edu

**Keywords:** photocatalytic reaction effectivity, photocatalytic reaction rate, nanoparticle sedimentation, titanium dioxide, methyl orange, reaction effectivity increasing

## Abstract

**Highlights:**

- Suspension depth: a new factor affecting photocatalytic reaction rate and effectiveness.
- Nanoparticle agglomeration is a factor decreasing the photocatalytic reaction rate.
- Photocatalytic reaction effectiveness increases by more than one order of magnitude with suspension depth reduction.

**Abstract:**

The influence of suspension depth and pollutant concentration on the rate and efficiency of photocatalytic degradation was investigated in aqueous TiO_2_ suspensions using methyl orange (MO) as a model pollutant. Both the reaction rate and the efficiency increased by more than one order of magnitude upon a relatively small decrease in suspension layer thickness. The reaction rate exhibited a complex N-shaped dependence on dye concentration, deviating from the monotonic behavior predicted by the Eley–Rideal and Langmuir–Hinshelwood mechanisms, as well as from the relationship derived in our previous study. To elucidate the origin of this behavior, nanoparticle aggregation was examined by sedimentation kinetics, low-acceleration centrifugation, and scanning electron microscopy (SEM). The results suggest that the interplay between enhanced dye adsorption and the reduction in the available photocatalyst surface area due to aggregation leads to the appearance of a maximum in the concentration dependence of the reaction rate. The relationship between reaction rate and photocatalytic efficiency was also analyzed. Although both parameters are correlated, efficiency values strongly depend on the selected reaction time interval, which complicates direct comparison between studies employing different experimental protocols. Consequently, the reaction rate appears to be a more reliable parameter for describing photocatalytic kinetics.

## 1. Introduction

The photocatalytic degradation of organic compounds is generally assumed to proceed according to one of two mechanisms: Langmuir–Hinshelwood [1,2,3,4,5] or Eley–Rideal [6]. Both mechanisms predict an increase in reaction rate with increasing pollutant concentration in the low-concentration range [1,6,7,8,9]. It has been shown that, within the concentration range of practical scientific interest (below 10 mM dm^−3^), the corresponding dimensionless concentrations remain very low [10].

However, several experimental studies involving titanium dioxide have demonstrated deviations from this expected behavior. During the degradation of methyl orange (MO), a maximum reaction rate was observed at 4 × 10^−5^ M over a concentration range of 2 × 10^−3^–1 × 10^−5^ M [11]. Similarly, maxima in the reaction rate were reported for propham degradation at 0.75 mM [12] and for gentian violet degradation at 0.25 mM [13]. In studies involving binary compounds, a reaction rate maximum for 4-chlorophenol photodecomposition was found at 30 µM [14]. Investigations of the photocatalytic degradation of 2-naphthol [15] and Reactive Yellow 145 [16] further showed that, after an initial increase with concentration, the reaction rate reached a plateau.

Similar effects have also been observed in systems without TiO_2_ photocatalysts. In the case of Congo red degradation using ZnO, a maximum degradation efficiency was reported at 30 ppm (43 µM dm^−3^) [17]. An even more complex N-shaped dependence, characterized by a maximum followed by a minimum, was observed during methylene blue photodegradation with ZnO [18].

Several explanations for these deviations from the predicted monotonic increase in reaction rate have been proposed. One hypothesis attributes the decrease at high pollutant concentrations to light absorption effects [19,20,21,22,23]. However, the model proposed in [18], which explicitly accounts for light absorption, still predicts a monotonic increase in reaction rate. Another explanation suggests that pollutant molecules and decomposition products block active sites on the photocatalyst surface, thereby suppressing radical generation [12,19,23,24,25]. Nevertheless, this mechanism cannot explain the secondary increase in reaction rate reported in [18]. It has also been suggested that, at low dye concentrations, diffusion toward catalytically active sites becomes limiting, leading to improved efficiency with increasing pollutant concentration [26].

In addition, a decrease in the reaction rate constant with increasing dye concentration, attributed to increased solution conductivity, has been reported [10]. Although this effect may contribute to the suppression of photodecomposition, it cannot account for the observed complex concentration dependence, since the decrease in the rate constant is substantially smaller than the simultaneous increase in reaction rate.

An alternative approach was proposed in [18], where the complex dependence of the reaction rate on organic concentration was explained by photocatalyst sediment covering the vessel bottom. The corresponding model qualitatively reproduced the experimentally observed N-shaped dependence. However, this interpretation also has limitations, as the experimentally observed maxima and minima are considerably sharper than those predicted theoretically. Furthermore, our recent results indicate that the complex dependence of photocatalytic reaction rate arises from the interplay of multiple simultaneous processes.

In previous work, a formula based on the Langmuir–Hinshelwood equation was derived by additionally accounting for light absorption and scattering within the suspension volume [18]. The calculated values showed good agreement with experimental data obtained for methylene blue degradation using ZnO [18] and methyl orange degradation using TiO_2_ [10]. The model reliably describes the dependence of reaction rate on suspension depth and photocatalyst loading. Nevertheless, it is less suitable for describing the correlation between reaction rate and methylene blue concentration, particularly in transparent suspensions. Therefore, it appears worthwhile to investigate the concentration dependence for other pollutants and photocatalysts.

One important conclusion derived from the model in [18] is that the reaction rate increases with decreasing suspension depth. Since the reaction rate correlates with the reaction efficiency represented by the “*Eff* parameter” (see Section 4.1), it is of interest to determine whether *Eff* decreases with increasing solution depth.

Photocatalysts can be applied either as nanopowders [27,28,29] or in immobilized form [30,31,32]. Nanosized photocatalysts generally provide substantially higher photocatalytic reaction rates [18,33,34]. However, nanopowder systems also introduce practical difficulties related to particle precipitation, catalyst removal, and accurate efficiency measurements. Importantly, the mechanisms proposed so far do not fully explain the occurrence of a maximum in reaction rate as a function of dye concentration. Therefore, the present study also examines the influence of model dye pollutants on photocatalyst precipitation, although there is a work that has suggested that dyes such as methyl orange and rhodamine B exert little practical influence on sedimentation behavior [35].

Several problems and their solutions should be discussed in the use of titanium dioxide for photocatalytic degradation. One of them is preventing the transformation of TiO_2_ phases during high-temperature calcination, which can be done with the help of geopolymers [36]. Another problem that should be addressed is expanding the spectral sensitivity into the visible light region, and improving the antimicrobial effect. This can be done by grafting the nanoparticles with biocidal materials [37]. Improving the photocatalytic activity of titanium dioxide can also be achieved by doping it with metal ions such as molybdenum, cobalt [38], platinum, silver [4], gold [5], and others.

The aims of the present work are (i) to check experimentally whether the photocatalytic degradation rate of MO with titanium dioxide correlates with the formula, described in [18], (ii) to investigate the reason for the deviation of this dependence from that predicted by the Langmuir–Hinshelwood model, Eley–Rideal model, and the formula obtained in [18], (iii) to investigate the dependence of reaction effectivity on the solution depth, and (iv) to investigate the sedimentation of the TiO_2_ nanopowder in MO solution.

## 2. Materials and Methods

### 2.1. Materials

Titanium dioxide powder (CAS 1317-70-0; Nano Arc anatase, 99.9% purity, average particle size 32 nm, specific surface area 45 m^2^ g^−1^; Thermo Fisher GmbH, Dreieich, Germany) was used as the photocatalyst. Methyl orange (MO; CAS 547-58-0, Valerus Ltd., Sofia, Bulgaria) solutions were prepared using double-distilled water (conductivity σ < 1 μS cm^−1^).

The initial dye concentrations were 5, 10, 20, 30, 40, 50, 60, 80, and 100 μM dm^−3^. Additional concentrations were occasionally employed to better define the trends and extrema. The concentration of titanium dioxide was fixed at 100 mg dm^−3^, except for scanning electron microscopy (SEM) analysis, where a lower concentration of 50 mg dm^−3^ was used to avoid very dense particle deposition on the substrate.

### 2.2. Reaction Rate and Efficiency Measurements

Experiments investigating the dependence of reaction rate and efficiency on suspension depth and pollutant concentration were conducted in a thermostated reactor equipped with a magnetic stirrer. The volume of the MO solution was maintained at 0.5 dm^3^. Vessels of various diameters were used to obtain different suspension depths.

The solution surface was illuminated at a 6000 lux intensity using a 300 W Hanau (Hanau, Germany) quartz lamp. The illumination was measured with a Mastech MS6610 (Shenzhen, China), luxmeter. The temperature was maintained at 293 K. A fixed time interval of 2 min was used to determine both reaction rate and efficiency.

Solution transparency was measured at 464 nm for low MO concentrations and at 520 nm for concentrations above 50 μM dm^−3^. All spectrophotometric measurements were performed using a Jasco V-670 UV–VIS (Hachioji, Japan) spectrophotometer. Before measurements, the photocatalyst was separated by centrifugation at 10,000× *g* for 40 min.

### 2.3. Sedimentation Investigation

The sedimentation experiments were conducted as follows: First, an MO solution of known concentration was prepared, and its transparency (T_1_) was measured. Titanium dioxide was then added at a concentration of n_init_, and the suspension was intensively stirred.

Subsequently, the suspension was stirred for 2 min under the same conditions as in the reaction rate and efficiency measurements. The suspension was then centrifuged at a low acceleration of 1000× *g* for 30 min. After centrifugation, the transparency (T) was measured, and the residual nanoparticle concentration n_res_ was determined.

A relatively low centrifugation speed (1000× g) was selected to allow for a more detailed observation of sedimentation processes.

### 2.4. Zeta Potential Measurements

An attempt was made to estimate particle size and aggregation behavior in MO solutions using a Zetasizer Nano ZS (Malvern Panalytical, Malvern, UK). However, the results were deemed unreliable by the instrument software and are therefore not reported. Consequently, nanoparticle aggregation was assessed indirectly through sedimentation experiments and SEM observations.

### 2.5. SEM Analyses

A scanning electron microscope (SEM; EVO 10, Carl Zeiss GmbH, Oberkochen, Germany) was used to examine the aggregation of photocatalytic particles formed during the precipitation experiments. Imaging was performed in the secondary electron mode at a magnification of 15,000×.

In the initial experiments, the nanopowder concentration was 100 mg dm^−3^. Under these conditions, the SEM images were too dense, making the results difficult to interpret. Therefore, the photocatalyst concentration was reduced to 50 mg dm^−3^, as mentioned above.

The samples for microscopic observation of photocatalyst particle aggregation were prepared as follows: Photocatalyst was added at a concentration of 50 mg dm^−3^ to solutions containing the corresponding MO concentrations (0, 20, 30, 40, 50, 60, 70, and 100 µmol dm^−3^). After intensive initial stirring, the suspension was stirred for an additional 2 min using a magnetic stirrer under the same conditions as those used for measuring the reaction rate and the parameter *Eff*. A drop of the resulting suspension was placed onto a mechanically polished zinc plate (polished with diamond paste to a particle size of up to 0.5 µm), and the liquid was allowed to evaporate.

## 3. Results and Discussion

Many factors influence the rate of the photocatalytic reaction: amount of catalyst, concentration of pollutant, temperature, light intensity, pH [39,40], scavengers [41], and others. Most substances used as scavengers slow down the reaction, but there are some, such as hydrogen peroxide [42], that accelerate it. In the following paragraphs, only a few of these factors are discussed.

### 3.1. Dependence of the Reaction Rate on the Initial Dye Concentration

**Figure 1:**
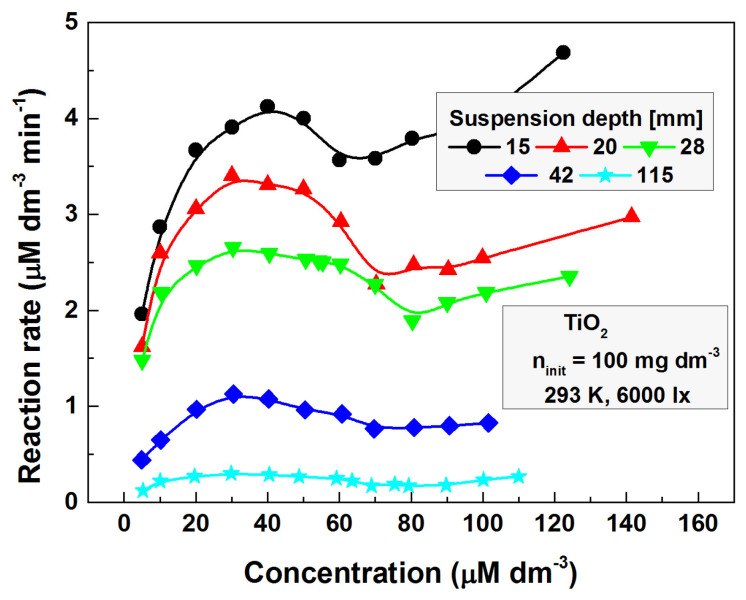
Dependence of the reaction rate on MO concentration for different suspension depths (*d*). The centrifugation acceleration is 10,000× *g*.

The dependence of the reaction rate on the initial MO concentration at different solution depths is shown in Figure 1.

As shown in Figure 1, the reaction rate initially increases with increasing dye concentration, reaches a maximum, and then decreases. The dependence is N-shaped, since after passing through a minimum, the reaction rate increases again. The overall shape and the corresponding values are very similar to those reported in [18], although both photocatalyst and pollutant differ in the present study. The extrema are less pronounced than those reported in [18] but are considerably sharper than those predicted by the model proposed in this work (i.e., ref. [18]). In addition, that hypothesis cannot be applied here, because the solutions used in the present study are much less transparent than those used in [18]. The maximum occurs at (31.1 ± 1.1) µmol dm^−3^ and appears to be independent of solution depth. This concentration is close to the values reported in [11,14].

The minimum is observed at approximately 60 µmol dm^−3^ for small solution depths and shifts slightly toward 80 µmol dm^−3^ at greater depths. Evidently, the dependence of the reaction rate on concentration cannot be described by Equation (8).

### 3.2. Dependence of the Reaction Rate on the Solution Depth

**Figure 2:**
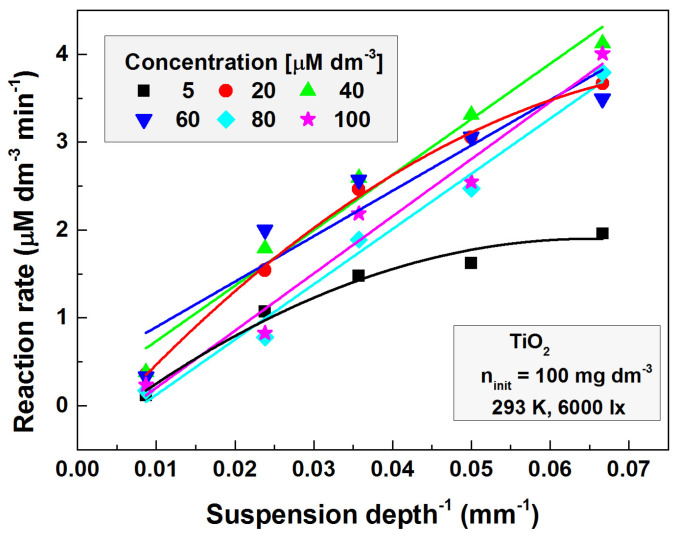
Dependence of the reaction rate on suspension depth for different MO concentrations. Photocatalyst concentration: 100 mg dm^−3^; T = 293 K; illumination intensity, E = 6000 lx. The centrifugation acceleration is 10,000× *g*. Solid lines are fitted to the experimental points according to Equation (8).

The dependence of the reaction rate on the parameter *1/d* for a catalyst concentration of 100 mg dm^−3^ and different MO concentrations is presented in Figure 2.

As expected from Equation (8), the reaction rate increases as the suspension depth decreases, and the increase exceeds one order of magnitude. This dependence becomes more linear with increasing MO concentration because the contribution of the exponential term in the numerator of Equation (8) decreases.

Based on the present observations and the findings reported in [18], it can be concluded that the concentration dependence predicted by Equation (8) is not valid for the photodegradation of MO in the presence of titanium dioxide, nor for highly transparent methylene blue solutions in the presence of zinc oxide.

The initial increase in reaction rate with increasing pollutant concentration may be attributed to the larger number of pollutant molecules adsorbed onto the catalyst surface. One possible explanation for the subsequent decrease is the aggregation of catalyst particles. If the organic molecules act as an aggregation agent, the active catalyst surface area decreases at higher dye concentrations. The competition between enhanced pollutant adsorption and the reduction in available photocatalyst surface area may contribute to the appearance of a maximum in the concentration dependence of the reaction rate.

### 3.3. Sedimentation

Titanium dioxide is one of the most common nanomaterials found in aquatic environments [43]. For this reason, its sedimentation behavior has been widely discussed in the literature [35,44,45,46,47,48,49]. Most studies on the aggregation and coagulation of TiO_2_ nanoparticles focus on their removal from various types of water, including seawater, lagoon water, river water, groundwater, and surface water. The influence of various factors, such as pH, coagulant addition, and organic matter content, has been examined. In the present work, attention was focused on the dependence of sedimentation on centrifugation acceleration, centrifugation time, and organic pollutant concentration.

#### 3.3.1. Sedimentation Kinetics

**Figure 3:**
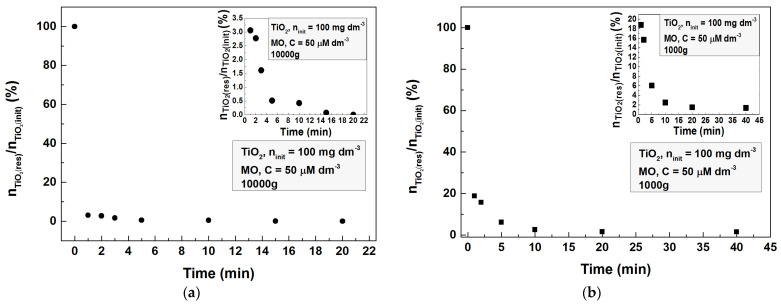
Dependence of the residual nanopowder amount on the time of centrifugation for acceleration: (**a**) 10,000× *g*; (**b**) 1000× *g*.

The dependences of the residual photocatalyst amount on centrifugation time at accelerations of 10,000× *g* (Figure 3a) and 1000× *g* (Figure 3b) were recorded. At both centrifugation speeds, the residual nanopowder concentration decreases sharply during the first minute. By comparing the sedimentation dynamics of titanium dioxide particles (Figure 3) with the dependences reported in [50], it may be assumed that the real particle size is significantly larger than the initial size specified by the manufacturer. The most probable explanation is nanoparticle aggregation.

From the figure, it can be seen that at 1000× *g*, the residual amount does not reach zero (Figure 3b), whereas at 10,000× *g*, it becomes practically negligible after approximately 20 min (Figure 3a). Therefore, centrifugation at 10,000× *g* was used to study the dependence of reaction rate and efficiency, whereas centrifugation at 1000× *g* was used to investigate sedimentation behavior.

**Figure 4:**
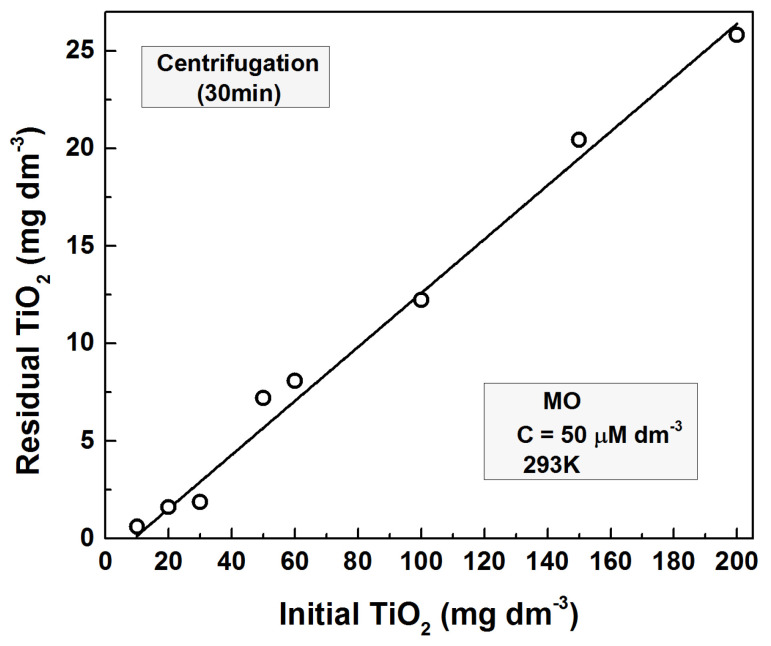
Dependence of the residual TiO_2_ nanopowder amount on the initial TiO_2_ amount after 30 min of centrifugation in a 50 µmol dm^−3^ MO solution. The centrifugation acceleration is 1000× *g*.

Figure 4 shows the dependence of the residual TiO_2_ amount on its initial amount. The respective correlation is clearly linear and can be described by Equation (1):

(1) $$n_{res}=0.1381n_{init}-1.23,$$

where *n_res_* is the residual amount of photocatalyst after centrifugation, and *n_init_* is its initial amount. The values calculated according to Equation (1) are shown in the figure as a solid line.

Sedimentation of nanoparticles may either increase or decrease with increasing nanoparticle concentration [51]. In the present case, it remains essentially constant within the investigated range of photocatalyst concentrations, with an average of (88.0 ± 7.0)% of the nanopowder precipitated.

#### 3.3.2. Dependence of the Sedimentation on the Dye Concentration

**Figure 5:**
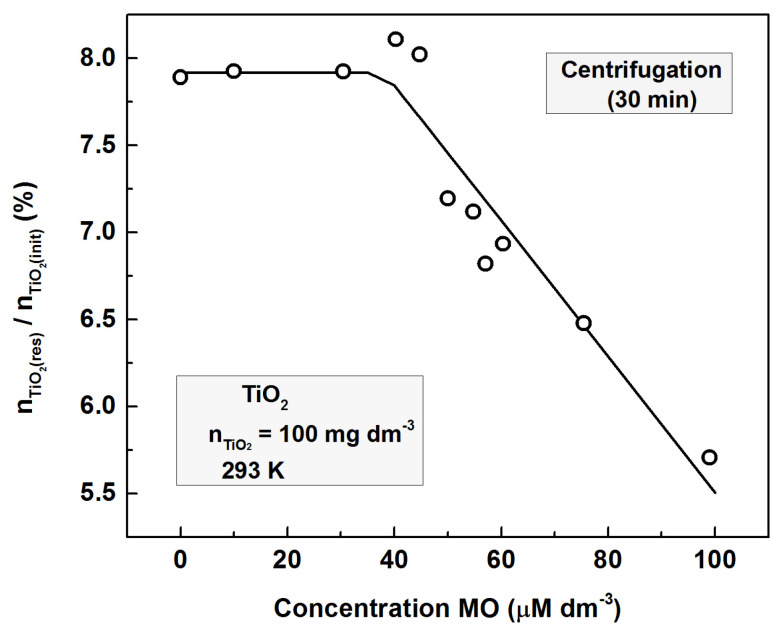
Dependence of the residual TiO_2_ amount after centrifugation on MO concentration. Initial TiO_2_ concentration: 100 mg dm^−3^; T = 293 K; acceleration is 1000× *g*.

The dependence of the residual titanium dioxide amount on methyl orange concentration is presented in Figure 5. This is the most important result of the sedimentation study represented in the present paper.

The sharp decrease in the residual titanium dioxide concentration at around 40 µmol dm^−3^ is probably due to coagulation of the aqueous suspension, with methyl orange acting as a coagulant. This result contrasts with the assumption in [43], according to which the presence of organic matter stabilizes aqueous suspensions and suppresses aggregation. The curve is similar to those reported for the coagulation of polychlorinated dibenzo-*p*-dioxins (PCDDs) and polychlorinated dibenzofurans (PCDFs) with ferric chloride [52] and for the precipitation of potassium hydroxide and potassium carbonate by aluminum sulfate [53]. Consequently, the assumption for aggregation of photocatalyst particles with increasing dye concentration may explain the sharp decrease in photocatalytic degradation rate observed above a certain MO content.

#### 3.3.3. SEM Observations

**Figure 6:**
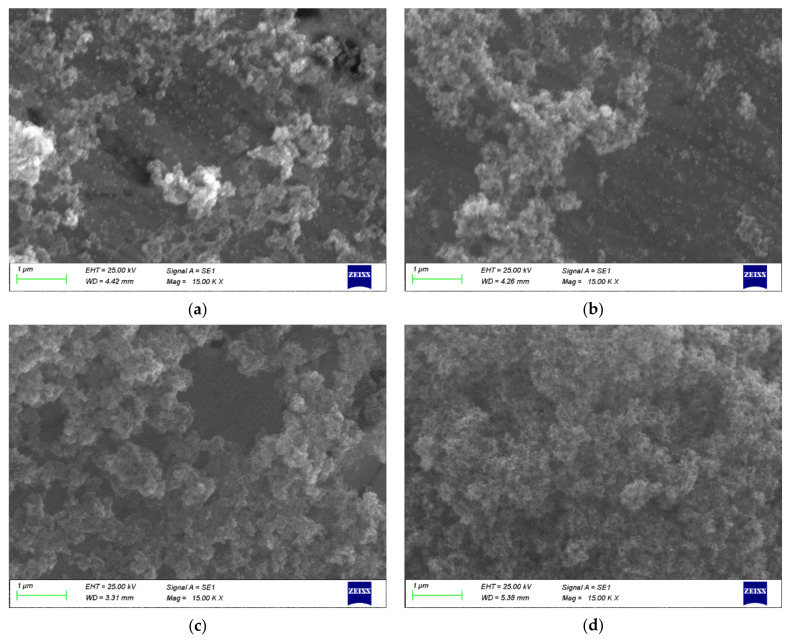
TiO_2_ nanoparticles after 2 min of agitation in an MO solution with concentrations of (**a**) 20 µM dm^−3^; (**b**) 30 µM dm^−3^; (**c**) 40 µM dm^−3^; (**d**) 60 µM dm^−3^. The centrifugation acceleration is 1000× *g*.

According to the literature [43], the presence of organic matter stabilizes aqueous suspensions and suppresses aggregation. However, the results presented in Section 3.3.1 and Section 3.3.2 indicate aggregation of photocatalyst particles, driven by the MO model pollutant. Therefore, independent experimental confirmation of this aggregation was required. Attempts to verify this by zeta potential measurements were unsuccessful, because the instrument software classified the data as unreliable. Consequently, SEM images of the photocatalyst particles were acquired after exposure to the same conditions as those used for reaction rate and efficiency measurements. The results support the assumption that titanium dioxide coagulates in the presence of methyl orange. Selected images are presented in Figure 6.

Small aggregates are visible in the SEM image (Figure 6a) of the nanopowder treated in a 20 µmol dm^−3^ solution. In addition, individual particles can still be observed, indicating that aggregation remains limited. In the case of a 30 µmol dm^−3^ solution, the accumulation of photocatalyst particles is clearly more pronounced, and the aggregates are larger. Individual particles are still present, albeit in lower numbers (Figure 6b). The aggregate size increment tendency at higher model pollutant concentrations is also obvious in Figure 6c,d. Further, individual photocatalyst particles are no longer observed at concentrations of 40 and 60 µmol dm^−3^. Thus, the SEM observations clearly confirm that the photocatalyst nanoparticles aggregate and that MO acts as a coagulant.

### 3.4. Dependence of the Reaction Effectivity on the Dye Concentration

**Figure 7:**
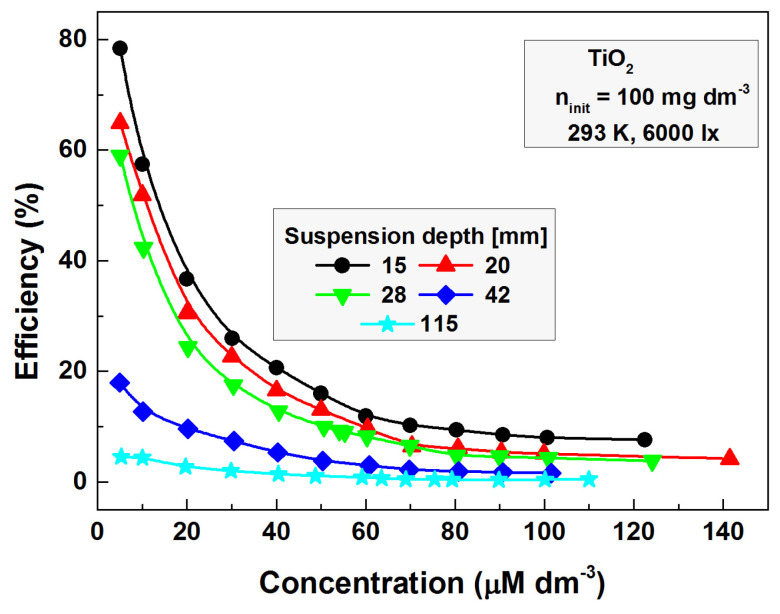
Dependence of reaction efficiency on MO concentration for different suspension depths. The centrifugation acceleration is 10,000× *g*. The experimental points are fitted by solid lines according to Equation (2).

There is no generally accepted standard method for evaluating the efficiency of photocatalytic processes in the literature. Efficiency may be evaluated by the percentage of substance degraded per unit time [54,55,56]. A considerable number of researchers express degradation efficiency using the *Eff* parameter (see Section 3.1). The dependence of this parameter on the initial MO concentration is shown in Figure 7.

As expected, *Eff* decreases with an increasing initial dye concentration, in agreement with observations reported by other researchers [25,57,58,59,60].

### 3.5. Dependence of the Reaction Efficiency on Solution Depth

**Figure 8:**
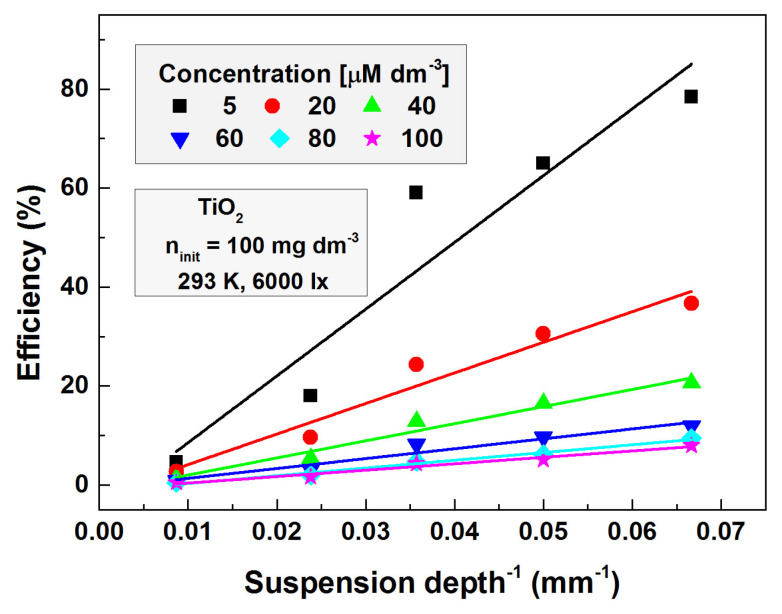
Dependence of reaction efficiency on suspension depth for different MO concentrations. The centrifugation acceleration is 10,000× *g*. The experimental points are fitted with solid straight lines.

It is of particular interest to determine whether the dependence of reaction rate on suspension depth, predicted by Equation (8), is also valid for the photodecomposition efficiency. The dependence of *Eff* on the parameter *1/d* is shown in Figure 8.

Decreasing the solution depth increases *Eff*. The dependence on *1/d* is almost linear, and the reaction efficiency increases by more than one order of magnitude. This result is important for the design of reactors for the photodegradation of organic substances: the smaller the depth of the pollutant suspension, the higher the degradation efficiency.

## 4. Theoretical Background

### 4.1. Degradation Efficiency

The degradation efficiency is determined as in Equation (2) [7,9,17,21,25]:

(2) $$Eff=\frac{100(C_{0}-C_{t})}{C_{o}}$$

where *C*_0_ is the initial concentration of the pollutant, and *C_t_* is the concentration value reached after a time *t*. The *C_t_* values can be determined by Equation (3):

(3) $$C_{t}=C_{0}-\int_{0}^{\tau }r\left(C_{0},t\right)dt$$

where *τ* is the chosen time interval and *r* is the reaction rate. Substituting (3) in (2) gives Equation (4):

(4) $$Eff=\frac{100(C_{0}-C_{0}-\int_{0}^{\tau }r\left(C_{0},t\right)dt)}{C_{0}}$$

or Equation (5):

(5) $$Eff=\frac{100\int_{0}^{\tau }r\left(C_{0},t\right)dt}{C_{0}}$$

Obviously, there is a correlation between Eff and reaction rate *r*.

The time interval *τ* in Equations (3)–(5) could be chosen with different durations, such as 1.5 h [25,26], 2 h [61], 2.5 h [57,62], 3 h [58,63], 3.5 h [64], and so on. That is why the value of the Eff parameter depends on the chosen period of measurements. If the chosen time is long enough, the Eff could be 100% even with highly varying other conditions of the experiment (pollutant concentration, temperature, amount of photocatalyst, etc.). That is why the reaction rate seems to be a more accurate criterion for the description of the photocatalytic degradation process.

It is interesting to discuss the maximum of the reaction effectiveness, described in [17]. If there is an extremum for some value of *C*_0_, then the derivative of Equation (5) must be zero (Equation (6)):

(6) $$\frac{dEff}{{dC}_{0}}={(C}_{0}\frac{d\int_{0}^{\tau }r\left(C_{0},t\right)dt}{{dC}_{0}}-1\int_{0}^{\tau }r\left(C_{0},t\right)dt)/C_{0}^{2}=0$$

That is possible only in the case where the numerator in Equation (6) equals zero, resulting in Equation (7):

(7) $$C_{0}\frac{d\int_{0}^{\tau }r\left(C_{0},t\right)dt}{dC_{0}}=\int_{0}^{\tau }r\left(C_{0},t\right)dt$$

But $r\left(C_{0},0\right)=C_{0}$. Then, $r\left(C_{0},t\right)=C_{0}U(t)$, where *U(t)* is a monotonously decreasing function of *t* and cannot possess a maximum for t>0. It seems that Eff could not possess an extremum for any *C*_0_ higher than zero.

### 4.2. Dependence of the Reaction Rate on the Catalyst Amount, Solution Depth, and Initial Pollutant Concentration

In our previous research [18], an equation was derived based on the Langmuir–Hinshelwood mechanism and the Beer–Lambert law. This equation can be written as follows (Equation (8)):

(8) $$r=\frac{kKnC(1-e^{-\left(lC+µn\right)d})}{\left(1+KC\right)\left(lC+µn\right)d}$$

where *r* is the reaction rate, *k* is the reaction rate constant, *C* is the concentration of the degradation compound, *K* is the adsorption equilibrium constant, *n* is the amount of the added photocatalyst, *μ* is the coefficient of absorption/scattering of light by the powder, *d* is the depth of the solution, and *l* is the coefficient of absorption of light by the pollutant.

### 4.3. Evaluation of the Residual Amount of TiO_2_

From the Beer–Lambert law applied for the solution of an absorbing compound in a non-absorbing solvent, for MO in water, it could be assumed that (Equation (9)):

(9) $$T_{1}=e^{-\mu_{1}Cd}$$

where $T_{1}$ is the solution transparency, *μ*_1_ is the absorption coefficient of MO, *d* is the thickness of the solution layer, and *C* is the solution concentration. In the same way, for the water suspension of TiO nanoparticles (Equation (10)):

(10) $$T_{2}=\;e^{-\mu_{2}nd}$$

where *μ*_2_ is the coefficient of absorption/scattering of TiO_2_ and *n* is the amount of nanopowder in mg dm^−3^.

If a nanopowder is loaded into an MO solution, the transparency of the resulting suspension *T* could be calculated by Equation (11):

(11) $$T=e^{-\mu_{1}Cd}e^{-\mu_{2}nd}=T_{1}T_{2}$$

If a certain nanoparticle amount *n_init_* is loaded into an MO solution with transparency *T*_1_, after centrifugation, the transparency will reach a value, assigned as *T*. Hence, the combination of Equations (9)–(11) results in Equation (12), suitable for residual TiO_2_ amount determination:

(12) $$n_{res}=\frac{-ln(\frac{T}{T_{1}})}{\mu_{2}d}$$

By dividing by *n_init_*, the residual TiO_2_ amount can be obtained in normalized form.

The values of *μ*_1_ and *μ*_2_ are determined from the calibration curves.

## 5. Conclusions

The present study examined the influence of methyl orange (MO) concentration and suspension depth on the photocatalytic degradation kinetics and efficiency in aqueous TiO_2_ suspensions, together with the sedimentation behavior of the photocatalyst. Sedimentation phenomena were investigated by kinetic measurements, low-speed centrifugation, and SEM analysis.

The main findings can be summarized as follows:

- The photocatalytic efficiency increases markedly with decreasing suspension depth. A reduction in the liquid layer thickness by less than one order of magnitude leads to an increase in the *Eff* parameter by more than one order of magnitude. Furthermore, the dependence of *Eff* on *1/d* is close to linear.
- The dependence of the reaction rate on methyl orange concentration exhibits a complex N-shaped behavior characterized by a maximum followed by a minimum. The maximum reaction rate occurs at 31.1 ± 1.1 µmol dm^−3^ and is practically independent of suspension depth. In contrast, the minimum shifts from approximately 60 µmol dm^−3^ at small depths to about 80 µmol dm^−3^ at larger depths. Unlike the reaction rate, the photocatalytic efficiency decreases monotonically with an increasing dye concentration.
- Decreasing the suspension depth by less than one order of magnitude results in an increase in reaction rate exceeding one order of magnitude. The relationship between reaction rate and *1/d* becomes progressively more linear at higher dye concentrations, which is attributed to the diminishing contribution of the exponential term in the numerator of the equation derived in our previous study and discussed herein.
- The experimentally observed dependence of the reaction rate during MO photodegradation in the presence of TiO_2_ differs from that predicted in the previous study. The most plausible explanation is aggregation of the photocatalyst particles induced by the dye, which acts as an aggregation-promoting agent.
- The sedimentation experiments further support the occurrence of nanoparticle aggregation. Within the investigated concentration range, increasing the TiO_2_ amount neither significantly enhances nor suppresses sedimentation. However, the residual amount of suspended photocatalyst decreases sharply at approximately 40 µmol dm^−3^ MO, indicating that the observed reduction in photocatalytic activity is associated with azo dye-induced aggregation of TiO_2_ nanoparticles.
- The reaction rate and the efficiency expressed by the *Eff* parameter are correlated. The dependence of *Eff* on the initial pollutant concentration could not exhibit extrema at concentrations above zero.
- The reaction rate appears to be a more suitable parameter for the description of reaction kinetics than the widely used *Eff* parameter.

## Data Availability

The original contributions presented in this study are included in the article. Further inquiries can be directed to the corresponding author.
